# Utilization of Algal Biochar for Biopassivation of Copper Sulfide Tailings to Reduce Acid Mine Drainage

**DOI:** 10.3390/biology14030300

**Published:** 2025-03-16

**Authors:** Zhiyuan Peng, Can Liu, Yuhang Fu, Hongwei Liu, Hongchang Liu, Hongpeng Cao

**Affiliations:** 1 School of Minerals Processing and Bioengineering, Central South University, Changsha 410083, China; pengzhiyuan@csu.edu.cn (Z.P.); liucan11212001@163.com (C.L.); 245611016@csu.edu.cn (Y.F.); hchliu2050@csu.edu.cn (H.L.); hongpengcao@csu.edu.cn (H.C.); 2 Key Laboratory of Biometallurgy, Ministry of Education, Changsha 410083, China

**Keywords:** algal biochar, copper sulfide tailings, acid mine drainage, biopassivation, environmental remediation

## Abstract

Acid mine drainage (AMD) has serious impacts on the environment. To inhibit the generation of AMD from copper sulfide tailings at the source, in this paper, a strategy is developed for promoting the biopassivation of copper sulfide tailings using algal biochar, and the effects of the pyrolysis temperature and concentration of algal biochar on the passivation efficiency and stability are investigated. The results reveal that the introduction of algal biochar during the biopassivation of copper sulfide tailings significantly enhances the tailings passivation effect of the tested *Acidithiobacillus ferrooxidans* strain and greatly stabilizes the formed passivation layer. Algal biochar prepared with a pyrolysis temperature of 300 °C and applied at a concentration of 6 g/L not only optimizes biopassivation but also significantly improves the stability of the passivation layer. The complex mechanisms of algal biochar in this system include regulating the pH and oxidation‒reduction potential of the reaction system, effectively adsorbing microbial cells, efficiently aggregating metal cations in solution, stimulating the synthesis of extracellular polymeric substances, and accelerating electron transfer. This research offers a novel method for the benign treatment of copper sulfide tailings and resource utilization of algae.

## Simple Summary

This research proposes an eco-friendly method using algal biochar to mitigate acid mine drainage (AMD) from copper sulfide tailings. By optimizing biochar preparation temperatures (300 °C) and application concentrations (6 g/L), this study demonstrates enhanced microbial activity and durable protective barrier formation on tailings. The biochar functions through synergistic mechanisms: neutralizing acidity, immobilizing toxic metals via adsorption, and stimulating organic substance production that accelerates mineral layer development. This dual-action approach effectively inhibits mineral dissolution and acid generation while maintaining stable performance. The solution offers a cost-effective strategy for controlling mining waste pollution and repurposing algal biomass for environmental protection. Implementation could significantly reduce long-term contamination risks near mining sites, benefiting both ecosystems and adjacent communities through sustainable AMD prevention. The method’s practical scalability and multi-functional remediation mechanisms position it as a promising alternative to conventional AMD management techniques.

## 1. Introduction

In recent years, as copper mine resources have been developed and utilized, large amounts of copper sulfide tailings have accumulated. If not managed appropriately, they will lead to severe environmental pollution. Sulfide copper tailings from open piles undergo oxidation due to the combined effects of water, oxygen, and microorganisms, leading to the generation of wastewater with high acidity, elevated sulfate levels, and significant heavy metal concentrations, commonly referred to as acid mine drainage (AMD). High concentrations of heavy metal ions are the main causes of water and soil pollution. Simultaneously, it severely harms the local biotic community, resulting in a loss of biodiversity and causing severe impacts on the stability of ecosystems. In addition, emissions from AMD pose potential risks to human health. The reaction mechanism for the generation of acid mine drainage from copper sulfide tailings is as follows:

2CuFeS_2_(s) + 4O_2_(aq)→2Fe^2 +^ +2SO_4_^2−^ + Cu^2+^ (1)

4Fe^2+^ + O_2_(aq) + 4H^+^ → 4Fe^3+^ + 2H_2_O(aq) (2)

Fe^3+^ + 3H_2_O(aq) → Fe(OH)_3_(s) + 3H^+^ (3)

CuFeS_2_(s) + 16Fe^3+^ + 8H_2_O(aq) → 17Fe^2+^ + 16H^+^ + 2SO_4_^2−^ + Cu^2+^ (4)

Techniques for addressing AMD issues include both treatment and prevention. Certainly, from a long-term perspective, relying solely on treatment is not a fundamental solution; preventing the generation of AMD at its source is more critical than treating it after it has formed.

Since the formation of AMD requires the joint participation of oxygen, water, and microorganisms [13], the exclusion of any one of these factors can reduce or even prevent the occurrence of AMD. The techniques used to prevent AMD can be divided into three types: oxygen barrier methods, sterilization, and surface passivation. Among these methods, surface passivation has been widely applied because of its simplicity of operation and fewer restrictions. By adding suitable passivating agents, a stable and dense chemical protective layer can be formed on the surface of sulfide minerals, thus inhibiting acid production. However, existing passivators have limitations in terms of application. Consequently, additional management strategies that are more stable, cost-effective, and environmentally sustainable are needed.

The utilization of microbial mineralization to passivate tailings has become an emerging research direction in recent years. During the leaching of chalcopyrite, passivation occurs simultaneously [17]. Studies have shown that the passivation layers on the surface of chalcopyrite mainly consist of polysulfide, elemental sulfur, and insoluble sulfate (such as jarosite). In these layers, insoluble sulfate is considered the primary passivation substance. The formation mechanism of insoluble sulfate layers is as follows:

M^+^ + 3Fe^3+^ + 2SO_4_^2−^ + 6H_2_O → MFe_3_(SO_4_)_2_(OH)_6_ + 6H^+^ (M is K^+^, Na^+^, NH4^+^, H_3_O^+^) (5)

Pan et al. utilized *Acidithiobacillus ferrooxidans* to treat sulfide tailings, and 5 g of tailings yielded 2.545 g of a passivation layer (jarosite) after biopassivation. Furthermore, studies have shown that the addition of substances such as fly ash, humic acid, and biochar can further promote the process of biopassivation of chalcopyrite. Integrating these substances with the biological passivation process presents a novel strategy for improving the efficacy of biological passivation.

Algae are characterized by their rapid growth rate, strong environmental tolerance, and high efficiency in CO_2_ fixation, and are widely used in various fields [22,23], such as food, environmental, energy, and medical applications. However, in eutrophic water bodies, algae often proliferate rapidly, leading to severe environmental issues such as algal blooms and red tides. Compared with research on the applications of algae, there is relatively little research on effectively handling large amounts of algae residues or waste, and developing methods for the resource utilization of algae waste is an urgent need.

The utilization of algae as a feedstock for the production of biochar materials has become an emerging area of research in recent years. Owing to its large surface area, rich organic functional groups, and inorganic minerals, algal biochar has excellent adsorption properties and ion exchange capabilities. Compared with biochar derived from lignocellulosic sources, algae-based biochar has a lower carbon content but higher levels of nitrogen, phosphorus, and other nutrients. Furthermore, algal biochar exhibits a higher cation exchange capacity and a higher pH, which are beneficial for the remediation of acidic environments. Additionally, the unique functional groups on algal biochar further enhance its adsorption efficiency. Algal biochar has shown significant application potential in environmental remediation and is widely utilized for the improvement of contaminated soils, water resource restoration, air purification, and pollutant degradation.

Algal biochar is also related to AMD management in various ways. Algae are an important component of AMD bioremediation technology [38]. Biochar can regulate the pH of AMD while adsorbing metal ions and pollutants, restoring water and soil resources contaminated by AMD [39,40]. On the other hand, biochar can function as a passivator, neutralizing tailings and preventing the formation of AMD [41,42].

However, owing to the scarcity of related research, the impact and mechanism of action of algae biochar on the biopassivation process remain unclear. Yang et al. [43] made a preliminary exploration into the promotion of passivation by biochar; however, their research primarily focused on high-grade chalcopyrite during the bioleaching process rather than on tailings post-leaching. Consequently, it does not closely align with real-world application scenarios and does not delve into the subsequent acid production of the passivated ore samples. Furthermore, while Pan et al. [20] conducted simulated acid production experiments on bio-passivated tailings, the issue of the passivation layer’s insufficient stability still requires further optimization.

This study used *Limnospira maxima* as a raw material to produce biochar and combined the biochar with *Acidithiobacillus ferrooxidans* to passivate copper tailings; then, the passivation effect and stability of the treated materials were assessed. The objectives of this study are as follows: (1) To investigate the ability of algal biochar to promote the biopassivation of chalcopyrite; (2) to explore the effects of pyrolysis temperature and biochar concentration on the promotion of biopassivation by algal biochar. (3) To elucidate the mechanism by which algal biochar promotes biopassivation.

## 2. Materials and Methods

### 2.1. Strain Selection and Cultivation

*Acidithiobacillus ferrooxidans* ATCC 23270 (*A. ferrooxidans*) is a Gram-negative bacterium that is obligately aerobic and mesophilic and has an optimal growth temperature range of 20–40 °C. It is acidophilic, can grow in environments with a pH of 1.0–6.0, and is chemolithoautotrophic, obtaining energy for growth and metabolic activities by oxidizing Fe^2+^ or reducing sulfur compounds. The strain of *A. ferrooxidans* used in this study was provided by the Key Laboratory of Biometallurgy, Ministry of Education, Central South University.

First, 100 mL of 9K liquid medium was added to a 250 mL conical flask, 4 g of anhydrous ferrous sulfate was added, and the initial pH was adjusted to 2.0 using sulfuric acid. Bacteria were added at a 10% ratio, and the flask was placed in an air incubator shaker set at 30 °C and 180 r/min for cultivation. Samples were taken regularly, and cells were counted with an optical microscope until the cell concentration reached 6 × 10^7^ cells/mL or higher. This process was repeated three times to fully activate the strain.

### 2.2. Sample Preparation and Characterization

The chalcopyrite tailings samples utilized in this experiment were sourced from the low-grade copper ore bioleaching field at the Zijin Mountain copper mine in Longyan City, Fujian Province. The ore was crushed into small pieces using a jaw crusher and subsequently ground into powder form using a vibration mill. The powdered ore was then sifted through a 100-mesh sieve by a vibrating sieve machine. Given the extremely fine nature of a sample and its high susceptibility to oxidation in the air, the sifted sample was promptly transferred into a sealed bag for storage to prevent oxidation.

### 2.3. Preparation of Algal Biochar

The algal species *Limnospira maxima* FACHB-438 used in this experiment belongs to the phylum Cyanobacteria, family Treponema, and genus Spirulina. It was sourced from the Freshwater Algae Species Bank of the Institute of Hydrobiology, Chinese Academy of Sciences. The culture was expanded using an SP culture medium and subsequently prepared through filtering, washing, and freeze-drying. The dried cyanobacteria were then placed in a crucible and heated in a muffle furnace. Argon gas was continuously introduced to maintain an anaerobic environment within the furnace. The temperature was incrementally increased to 300 °C, 400 °C, and 500 °C at a rate of 5 °C per minute, and pyrolysis was performed at these set temperatures for 120 min each. Upon completion of pyrolysis, argon gas circulation was maintained until the temperature decreased to 80 °C. The products were allowed to cool naturally to below room temperature before the crucible was removed, and the contents were ground through a 100-mesh sieve. The resulting algal biochars produced at different pyrolysis temperatures were designated ABC300, ABC400, and ABC500.

### 2.4. Biopassivation Experiment

Biopassivation experiments were conducted in 250 mL conical flasks containing 100 mL of 9 K medium and 5 g of chalcopyrite tailings. Ferrous sulfate heptahydrate (7.5 g) was added to each flask, and the initial pH was adjusted to 2. Activated bacteria were added at a 10% ratio, and then the algal biochar was added according to the experimental requirements. The flasks were incubated in a constant-temperature shaker in air with a temperature set at 30 °C and a rotational speed of 180 r/min. Algal biochar prepared at different pyrolysis temperatures (ABC300, ABC400, and ABC500) was added to investigate the effect of biochar pyrolysis temperature on the passivation effect. Additionally, different concentrations of ABC300 (2 g/L, 4 g/L, 6 g/L, 8 g/L, and 10 g/L) were used to further explore this effect. Three parallel groups were set up for each experimental group, along with a blank control. Samples were periodically taken to determine the pH, redox potential, metal ion concentration, and cell concentration of the solutions. After the passivation process was complete, the mineral samples from each group were filtered and air-dried for subsequent experimental analysis.

### 2.5. Simulated Precipitation Experiment

Five grams of mineral sample from each group in the biological passivation experiment was placed into a funnel device for a small-scale simulated precipitation experiment. The leachate from each group was collected daily; the pH, redox potential, and metal ion concentration of the solution were measured; and the stability of the passivation layers was verified.

### 2.6. EPS Extraction

A total of 100 mL of 9K liquid culture was prepared in 250 mL conical flasks. Four grams of anhydrous ferrous sulfate was added, and the initial pH was adjusted to 2.0 using sulfuric acid. Then, 5 g of copper sulfide tailings was added, and activated bacteria were added at a 10% ratio. To the experimental group, 6 g/L ABC300 was added. Three parallel groups and a blank control were set up. The flasks were placed in a constant-temperature shaker in air set at 30 °C and 180 r/min for biopassivation. Eight days later, the samples in each group were allowed to settle for 1.5 h to separate the bottom slag. Two grams of the bottom slag was removed and resuspended in 10 milliliters of 0.9% saline solution. One gram of sterile glass beads with a diameter of 0.5 millimeters was added, and the sample was shaken on a vortex mixer for 10 min. Then, the sample was centrifuged at 3000 r/min for 1 min, the supernatant was decanted, and the cell concentration of the supernatant was determined under an optical microscope. Ten milliliters of sterile water was added to the slag in the centrifuge tube, which was shaken on a vortex mixer. The mixture was centrifuged, and the supernatant was decanted and examined under a microscope. These steps were repeated until no microorganisms were visible in the decanted supernatant under the optical microscope. All the supernatants from the above steps were collected, centrifuged at 10,000 r/min for 5 min, and transferred into sterile test tubes. The extracellular polymeric substance solution on the mineral surface was collected, and the protein and polysaccharide contents of the solution were determined.

### 2.7. Analytical Methods

ICP‒OES (SPECTROBLUE, Kleve, Germany, FMX 26) was utilized to determine the ion concentration in the solutions. The concentration of ferrous ions was detected using the o-phenanthroline spectrophotometric method. The polysaccharide concentration in EPS was determined via the phenol‒sulfuric acid method. The protein content was measured using a BCA assay kit (Thermo Fisher Scientific, Waltham, MA, USA), with bovine serum albumin (BSA) serving as the standard protein. The free cell concentration was measured under an optical microscope using a hemocytometer (CX31, 400× magnification, Olympus, Tokyo, Japan). SEM‒EDS (Quanta 650 FEG, Hillsboro, ON, USA) was employed to analyze the surface morphology of the mineral samples, XRD (PANalytical X’Pert PRO, Almelo, The Netherlands) was used to analyze the surface composition, and X-ray fluorescence (XRF) (PNAalytical ZETIUM, Almelo, The Netherlands) was used to analyze the elemental composition of the mineral samples. XPS (Thermo Fisher X, Waltham, MA, USA ESCALAB 250Xi) was used to analyze the Fe and S compounds on the surface of the mineral samples. FTIR (Thermo Scientific LS-50, Waltham, MA, USA) was utilized to analyze the functional groups on the biochar samples.

## 3. Results and Discussion

### 3.1. Original Ore Sample Composition

**Figure 1:**
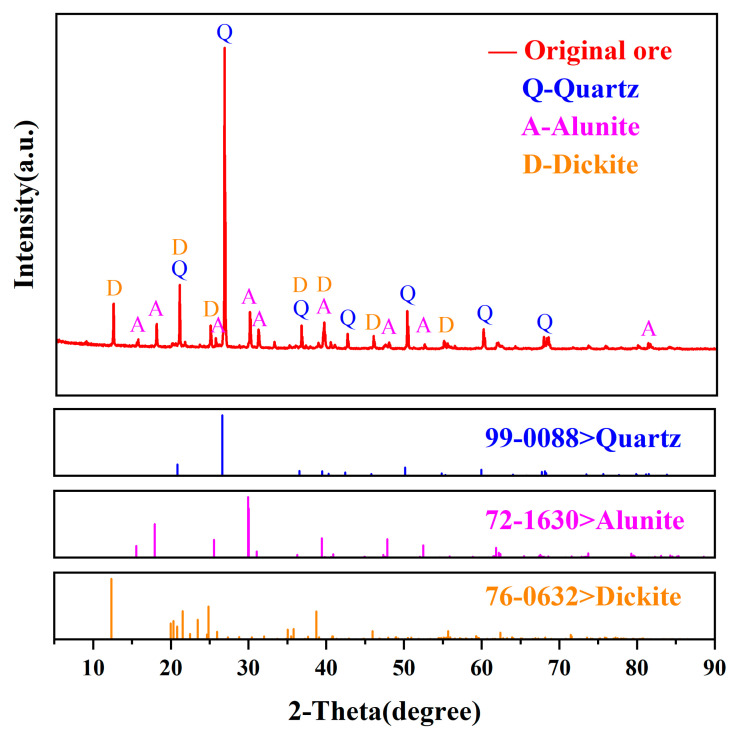
XRD pattern of the original ore sample.

The XRD results indicate that quartz accounted for a significant proportion of the experimental ore sample at 48.40%. The kaolinite content was 30.20%, and the alunite content was 21.40% (Figure 1).

The XRF results showed that the amount of Cu in the ore sample was only 0.1549%, that of Fe was 1.608%, and S was 6.141% (Table 1). The composition of the ore sample confirmed that the experimental material consisted of bioleached copper sulfide ore tailings.

### 3.2. FTIR Analysis of Algal Biochar Samples

**Figure 2:**
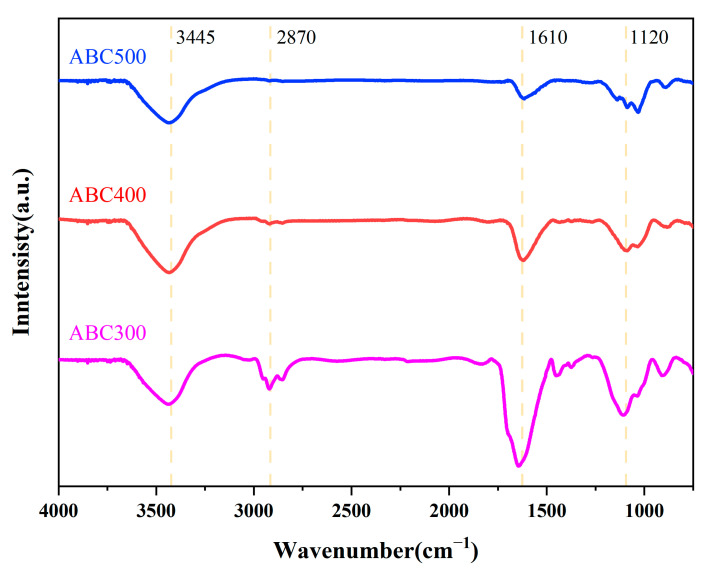
FTIR spectra of ABC300, ABC400, and ABC500.

Figure 2 presents the FTIR spectra of ABC300, ABC400, and ABC500. The absorption peak at 3445 cm^−1^ corresponds to the stretching vibration of -OH in alcohols, phenols, or carboxylic acids. The peak at 2870 cm^−1^ is attributed to symmetric C–H. The absorption peak at 1610 cm^−1^ is due to the stretching vibration of C=C in aromatics, as well as the stretching vibration of C=O in conjugated quinones or ketones. Finally, the peak at 1120 cm^−1^ is associated with the symmetric stretching of C-O-C in cellulose and hemicellulose ester groups [44]. As the pyrolysis temperature rose, the quantity of oxygen-containing functional groups, particularly C=O, in the algal biochar substantially diminished. It is widely accepted that biochar adsorption occurs primarily through five mechanisms: complexation, cation exchange, precipitation, electrostatic interactions, and chemical reduction. Notably, both complexation and cation exchange are dependent on the presence of oxygen-containing functional groups [45]. Consequently, owing to the presence of a greater number of oxygen-containing functional groups, ABC300 has superior adsorption capabilities for metal ions.

### 3.3. Biopassivation Experiment Results

#### 3.3.1. Effect of the Pyrolysis Temperature of Algal Biochar on the Effectiveness of Passivation

**Figure 3:**
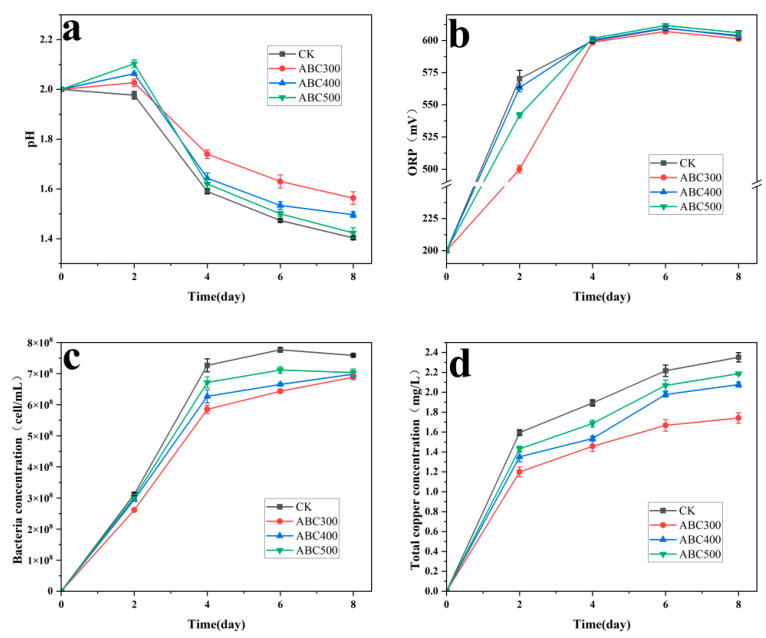
Changes in (**a**) pH, (**b**) ORP, (**c**) bacterial concentration, and (**d**) total copper concentration with different biochar addition amounts during the biopassivation experiment.

The pH of the experimental group clearly surpassed that of the control group, with the passivation system incorporating ABC300 resulting in the highest pH (Figure 3a). Studies have shown that at 30 °C, when the pH is less than 2.0, a higher pH is more conducive to the synthesis of jarosite [46]. This implies that algal biochar facilitates the creation of a passivation layer by increasing the pH of the biopassivation system.

The ORP of the solution increased within the first 4 days, with the experimental group exhibiting a slower ORP growth rate than the control group. The passivation system utilizing ABC300 displayed the slowest ORP growth rate (Figure 3b). Fe^3+^ was the primary oxidant in the system. Owing to the absence of a passivation layer in the early stages, the rapid accumulation of Fe^3+^ may have accelerated the dissolution rate of the tailings in the early part of the experiment [47], a phenomenon that can also be observed from the changes in total copper concentration in solution (Figure 3d). Consequently, algal biochar slowed the oxidation rate of Fe^2+^ in the early stages, inhibiting mineral dissolution. After 4 days, the ORP of all the groups stabilized above 590 mV, which was conducive to the formation of further jarosite passivation layers [48,49].

The concentration of free bacteria in the experimental groups was lower than that in the control group (Figure 3c), suggesting that algal biochar exerts a certain adsorption effect on *A. ferrooxidans*. Notably, the passivation system supplemented with ABC300 exhibited the lowest concentration of free bacteria, indicating that ABC300 has the strongest adsorption ability. When bioleaching microorganisms adhere directly to the surface of metal sulfide ores, they can catalyze the oxidation and decomposition of the ores, a process referred to as contact leaching [50]. Algal biochar prevents the direct oxidation of minerals by microorganisms by adsorbing numerous free cells, thus inhibiting the bioleaching of chalcopyrite tailings.

The rise in total copper concentration in the solution originated from the dissolution of the copper tailings. The total copper concentration in the experimental groups was notably lower than that in the control group, with the passivation system containing ABC300 exhibiting the lowest total copper concentration, the slowest growth rate, and the shortest time to stability (Figure 3d).

The experimental results indicate that algal biochar effectively inhibited the bioleaching of chalcopyrite tailings and promoted biological passivation. The results of the significance analysis clearly indicate that ABC300 substantially decreases the dissolution of copper ions and enhances the solution pH, showcasing the most effective passivation effect (Table 2). Nevertheless, as the pyrolysis temperature increases, this effect progressively diminishes.

#### 3.3.2. Effect of Algal Biochar Concentration on the Effectiveness of Passivation

**Figure 4:**
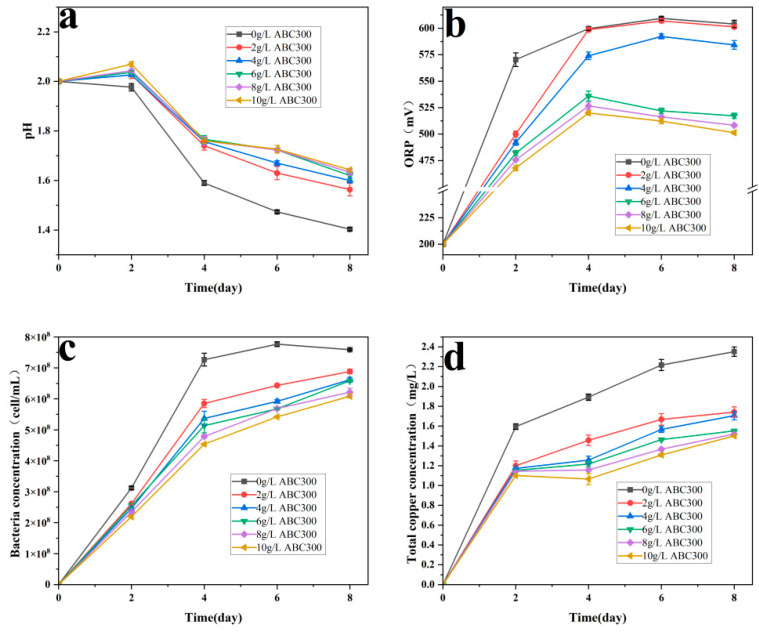
Changes in (**a**) pH, (**b**) ORP, (**c**) bacterial concentration, and (**d**) total copper concentration under different concentrations of biochar addition during the biopassivation experiment.

At low concentrations, as the concentration of ABC300 increased, the pH at the later passivation stage gradually rose. When the concentration of ABC300 reached 6 g/L, further increases in ABC300 concentration resulted in minimal changes in pH in the later passivation stage, reaching approximately 1.7 (Figure 4a), which is conducive to the formation of jarosite.

As the concentration of ABC300 increased, the rate of ORP increase gradually decreased. After the fourth day, for the groups with ABC300 concentrations at or above 6 g/L, the ORP began to decrease and eventually stabilized, with the ORP at stabilization also decreasing in sequence (Figure 4b). This indicates a significant transformation of Fe^3+^ into jarosite, which favors the formation of a passivation layer [48,49].

As the concentration of ABC300 increased, the concentration of free bacteria correspondingly decreased (Figure 4c), indicating that the adsorption effect of ABC300 on cells gradually increased with increasing concentration without affecting the growth rate of *A. ferrooxidans.*

As the concentration of ABC300 increased, the total copper concentration in each group gradually decreased. When the concentration of ABC300 reached 6 g/L or above, the total copper concentration postpassivation was essentially similar (Figure 4d).

We also measured the concentrations of ferrous and total iron in each group, but the change trends in the experimental and control groups were similar, with no significant difference. However, the total iron concentration at the final stage was the lowest in the passivation system with the addition of ABC300 (Appendix A). As the concentration of ABC300 increased, the total iron concentration gradually decreased by the end of passivation. This could be attributed to the further enhancement of electron transfer from sulfides to Fe^3+^ via surface protonation and hydration reactions due to the addition of biochar, resulting in the production of thiosulfate and the ultimate formation of jarosite [51,52].

The results of the significance analysis reveal that for the pH at the conclusion of the passivation experiment, when the concentration of ABC300 is less than 4 g/L, the pH rises notably with the increase in concentration. Upon reaching 4 g/L, the change in pH becomes insignificant. For the total copper concentration at the end of the passivation experiment, when the concentration of ABC300 is below 6 g/L, the total copper concentration decreases notably with the rise in concentration. Upon reaching 6g/L, the change in total copper concentration becomes insignificant (Table 3).

### 3.4. Simulated Precipitation Experiment Results

#### 3.4.1. Effect of Pyrolysis Temperature of Algal Biochar on the Stability of the Passivation Layer

Under the combined action of microorganisms, water, and oxygen, tailings from chalcopyrite generate AMD. This process results in the production of H^+^, Fe^2+^/Fe^3+^, and Cu^2+^, leading to a decrease in solution pH and an increase in ORP.

**Figure 5:**
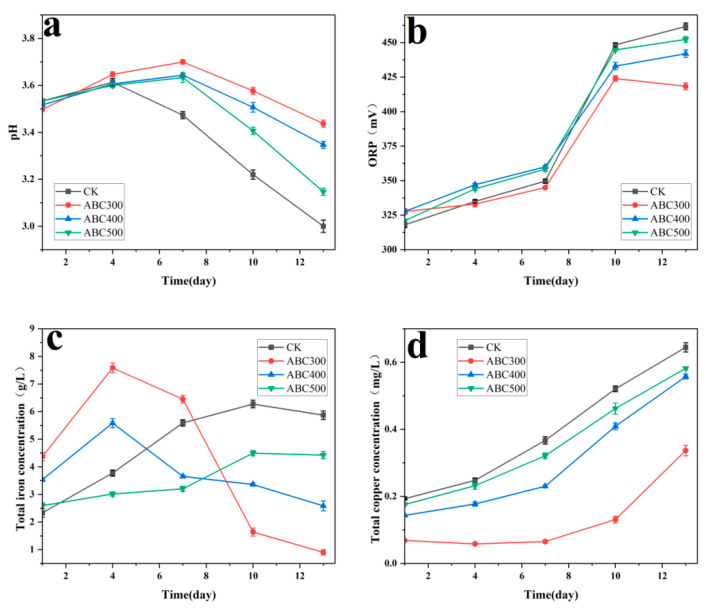
Changes in (**a**) pH, (**b**) ORP, (**c**) total iron concentration, and (**d**) total copper concentration of the leachate with different biochar addition amounts during the simulated precipitation experiment.

Initially, over the first 4–6 days, the pH of the leachate from each group increased to varying extents, followed by a subsequent decline until the conclusion of the experiment (Figure 5a). Notably, the control group presented the lowest pH and the most rapid rate of decrease, whereas the experimental groups exhibited a mitigated pH decrease. The leachate pH of the passivated mineral samples with ABC300 was the highest.

During the initial 10 days, the ORP of the leachates from all groups exhibited an upward trend. Beyond the 10-day mark, the ORP growth rate for the control group and the mineral samples passivated with ABC400 and ABC500 decelerated, whereas the ORP of the leachate from the mineral sample treated with ABC300 started to decrease and remained the lowest (Figure 5b).

The total iron concentration in the leachate in the control group continued to increase. Conversely, the total iron concentration in the samples treated with ABC300 and ABC400 began to decrease after four days (Figure 5c). Among these samples, the total iron concentration in the leachate of the passivated ore samples treated with ABC300 was the lowest.

Each group exhibited an increasing trend in total copper concentration; however, the experimental groups presented lower concentrations than the control group (Figure 5d). Notably, the leachate from mineral samples treated with ABC300 presented a markedly reduced total copper concentration relative to the other groups.

These results indicate that the addition of algal biochar effectively inhibited the dissolution of the passivation layer, enhancing its stability and thereby suppressing the generation of AMD. In the test materials, the leachate pH at the end of the experiment for the passivated ore sample treated with ABC300 was significantly higher than that of other groups, and the total copper concentration was significantly lower, demonstrating the best stability (Table 4).

#### 3.4.2. Effect of Algal Biochar Concentration on the Stability of Passivation Layers

**Figure 6:**
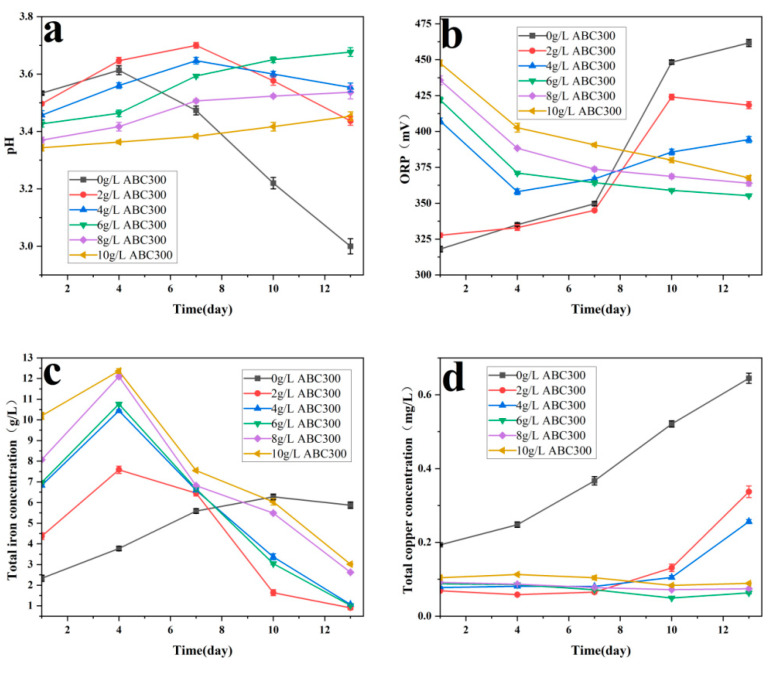
Changes in (**a**) pH, (**b**) ORP, (**c**) total iron concentration, and (**d**) total copper concentration of the leachate under different added concentrations of biochar during the simulated precipitation experiment.

When the concentration of ABC300 was low, the pH of the leachate slightly increased during the first 2–4 days and then continuously decreased. When the concentration of ABC300 reached 6 g/L or above, the pH of the leachate consistently increased. Additionally, with increasing ABC300 concentration, the initial pH of each group of leachates decreased (Figure 6a).

As the concentration of ABC300 increased, the initial ORP of each group of leachates increased. When the concentration of ABC300 was 2 g/L, the ORP of the leachate gradually increased and stabilized after 10 days. When the concentration of ABC300 reached 4 g/L or above, the ORP of the leachate consistently decreased (Figure 6b).

With increasing ABC300 concentration, the initial total iron concentration of each group of leachates increased. During the first 4 days, the total iron concentration of each group of leachates increased and then gradually decreased. The total iron concentration of groups with iron concentrations less than 6 g/L decreased to approximately 1 g/L after 14 days (Figure 6c).

When the concentration of ABC300 was below 4 g/L, the total copper concentration of the leachate remained essentially stable for the first 7 days but began to rise after 7 days. When the concentration of ABC300 reached 6 g/L or above, the total copper concentration of the leachate remained almost unchanged and consistently remained at a low level (Figure 6d).

The results of the significance analysis reveal that the pH of the leachate at the conclusion of the aforementioned experiments initially increased significantly and subsequently decreased with the escalation of ABC300 concentration, peaking at 6 g/L. Regarding the total copper concentration in the leachate, when the ABC300 concentration is below 6 g/L, the total copper concentration experiences a significant reduction as the concentration rises. Upon surpassing 6 g/L, the fluctuations in total copper concentration are not significant (Table 5).

In summary, algal biochar produced at a pyrolysis temperature of 300 °C markedly enhances the passivation effect and stability of the passivation layer in ore samples. However, as the pyrolysis temperature rises, the passivation effect and stability of the ore samples diminish, and the production cost of biochar escalates. When the concentration of ABC300 is under 6 g/L, the passivation effect and stability of the ore samples improve considerably with increasing concentration. Upon reaching 6 g/L, there is a marginal improvement, but it is not substantial. Consequently, from a practical application standpoint and taking into account the cost of raw materials and experimental outcomes, a concentration of 6 g/L of ABC300 is deemed the optimal condition for the biological passivation of copper sulfide tailings.

### 3.5. Characterization of Passivated Mineral Samples

#### 3.5.1. Surface Morphology and Elemental Composition of Ore Samples

**Figure 7:**
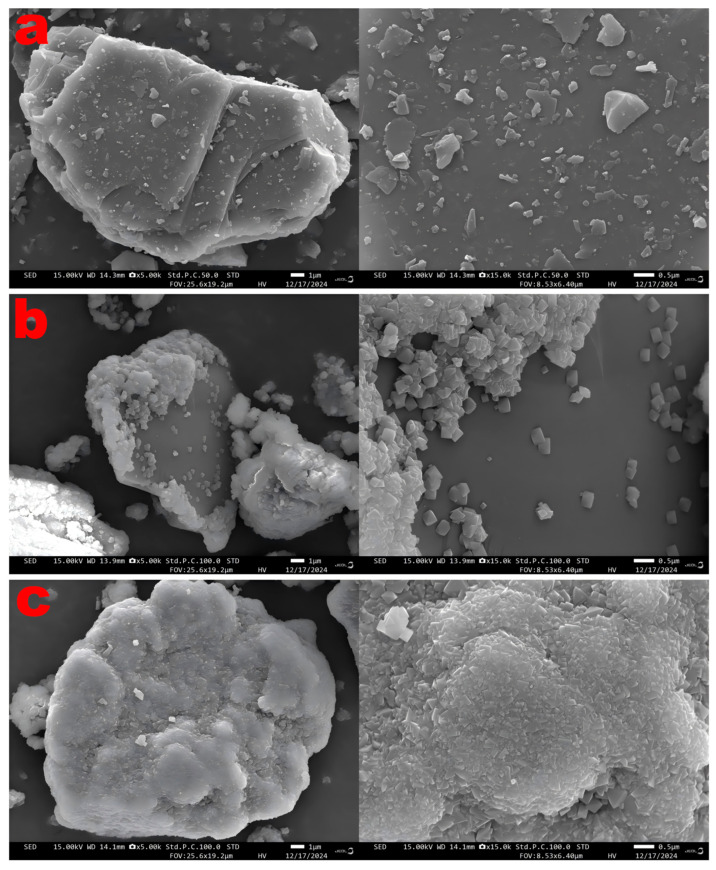
Scanning electron microscope images of ore samples before and after biopassivation: (**a**) original ore samples, (**b**) biopassivated ore sample without ABC300 addition, and (**c**) biopassivated ore sample with ABC300 addition.

Figure 7 shows the surface morphology of the chalcopyrite tailings before and after passivation. Prior to passivation, the tailings surface was irregular, featuring corrosion pits from leaching and sparsely distributed adhered particulate matter (Figure 7a). Following biological passivation, the mineral surface was enveloped by a multitude of regular cubic crystals, predominantly jarosite, which was also the primary component of the mineral surface passivation layer [50]. In the control group, the jarosite crystals adhered to the surface of the tailings samples were larger, unevenly distributed, and featured a thinner passivation layer, and part of the surface remained uncovered (Figure 7b). With the addition of ABC300, the jarosite crystals on the surface of the tailings samples became smaller, were closely arranged, had a thicker passivation layer, and completely enveloped the samples (Figure 7c).

The EDS results indicate that prior to passivation, the Fe content on the surface of the tailings sample was merely 0.35% (Table 6). However, following biological passivation, the Fe content on the surface of the ore samples increased substantially, with that in the control group reaching 17.49% (Table 7) and that in the experimental group reaching 38.57% (Table 8). Appendix B illustrates the distribution of each element on the surface of the ore samples. These results suggest that a significant amount of iron-containing substances were produced on the surface of the ore samples. Additionally, the Fe, S, and K contents on the surface of the passivated ore sample were elevated, consistent with the elemental composition of the jarosite.

The adsorption capacity of biochar can promote bacterial attachment and the enrichment of Fe^3+^ in solution, thereby enhancing the efficiency of electron exchange. Additionally, its porous structure not only prevents the aggregation of jarosite but also reduces its particle size [53]. Thus, the addition of algal biochar not only promotes the formation of a jarosite passivation layer but also enhances the structure of the passivation layer, making it denser and more uniform, thereby inhibiting the dissolution of tailings.

#### 3.5.2. Jarosite Content on the Surface of Ore Samples

**Figure 8:**
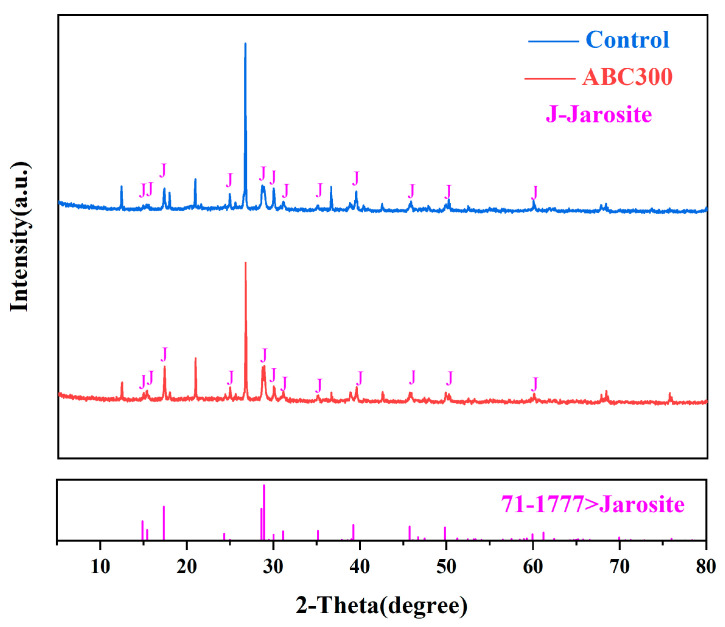
XRD patterns of the biopassivated ore samples.

Figure 8 shows the XRD patterns of the experimental and control groups after passivation. A comparison with the XRD pattern of the raw ore sample (Figure 1) reveals that the characteristic peaks of jarosite are present in the XRD patterns of the passivated samples. Furthermore, the characteristic peak intensity of pyrite in the experimental group was greater than that in the control group, indicating that the addition of biochar resulted in the formation of more jarosite on the mineral surface. Sulfides undergo oxidation via surface protonation and hydration reactions, transferring electrons to Fe^3+^ and thus producing thiosulfates. This process subsequently triggers the formation of jarosite, ultimately resulting in the passivation of tailings [51,52]. The addition of algal biochar promoted electron transfer during this process, thereby facilitating the formation of jarosite. These findings suggest that the biopassivation experiment effectively produced jarosite on the surface of the ore samples and that the inclusion of algal biochar enhanced the synthesis of jarosite, thus assisting in the passivation of the tailings.

#### 3.5.3. Forms of Fe and S on the Surface of the Ore Samples

**Figure 9:**
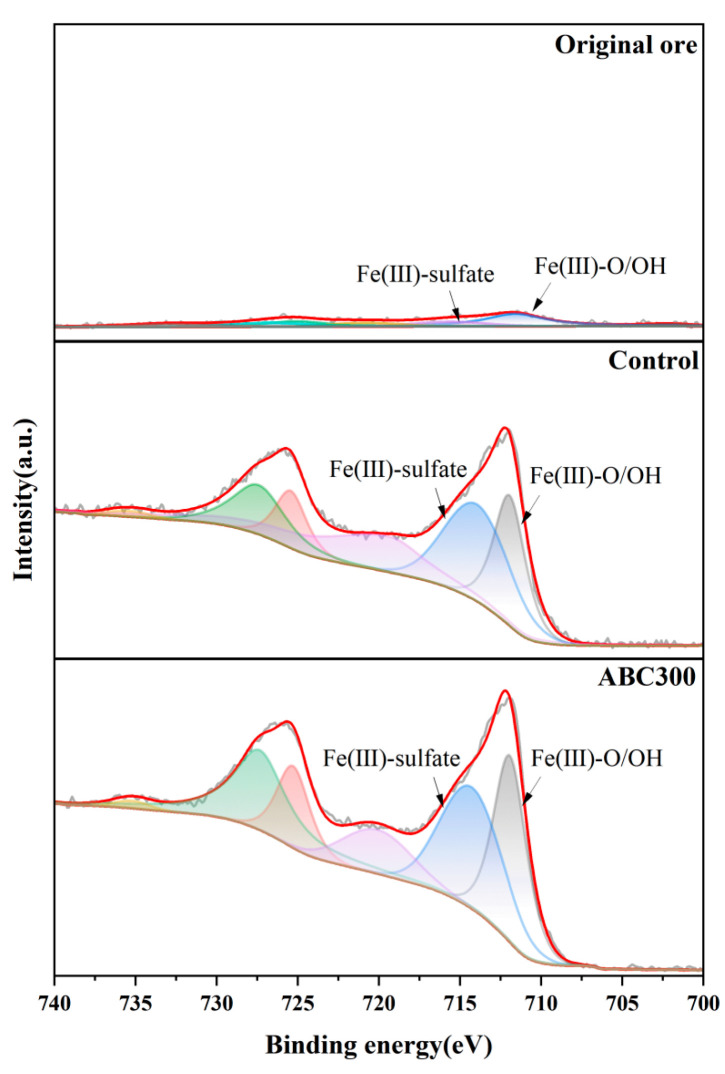
XPS spectra of Fe 2p of the original and biopassivated ore samples.

Figure 9 shows the Fe 2p spectra for the raw ore sample, the control sample, and the experimental sample. The peaks at a binding energy of approximately 712 eV correspond to Fe(III)-O/OH, whereas those at a binding energy of approximately 715 eV are indicative of Fe(III)-sulfate, suggesting the presence of jarosite in the samples. The Fe 2p spectrum of the raw ore sample exhibited a very low peak at 712 eV and nearly no peak at 715 eV. Following biopassivation, pronounced peaks emerged at both 712 eV and 715 eV, confirming the creation of jarosite. Moreover, the peaks of the experimental samples were higher than those of the control samples, suggesting that algal biochar further enhances the formation of jarosite.

**Figure 10:**
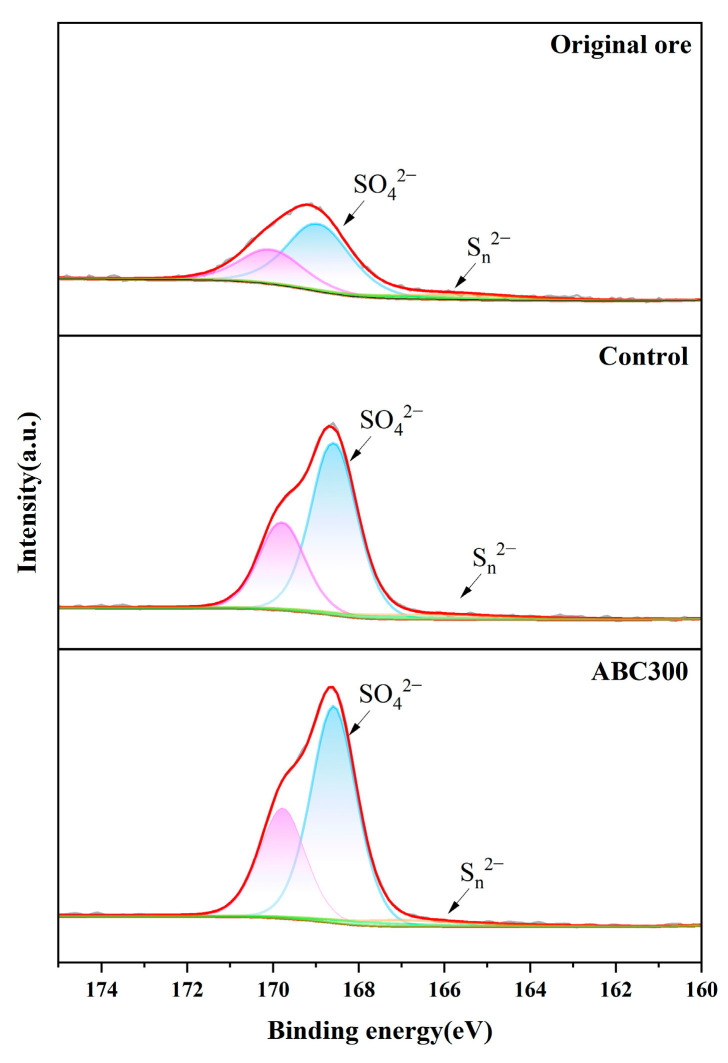
XPS spectra of S 2p of the original and biopassivated ore samples.

Figure 10 shows the S 2p spectra of the raw ore sample, the control sample, and the experimental sample. The peak at a binding energy of approximately 166 eV corresponds to S_n_^2−^ in polysulfides, while the peak at a binding energy of approximately 169 eV corresponds to SO4^2−^ in sulfates, predominantly jarosite. The S 2p spectrum of the raw ore sample exhibited lower peaks at 166 eV and 169 eV. Following biopassivation, a pronounced peak emerged at 169 eV, and the peak of the experimental sample was higher than that of the control sample, with a negligible peak at 166 eV. This suggests that the primary passivation substances are sulfates, specifically jarosite, and that the introduction of algal biochar facilitates the creation of jarosite.

### 3.6. Protein and Polysaccharide Contents in EPS

**Figure 11:**
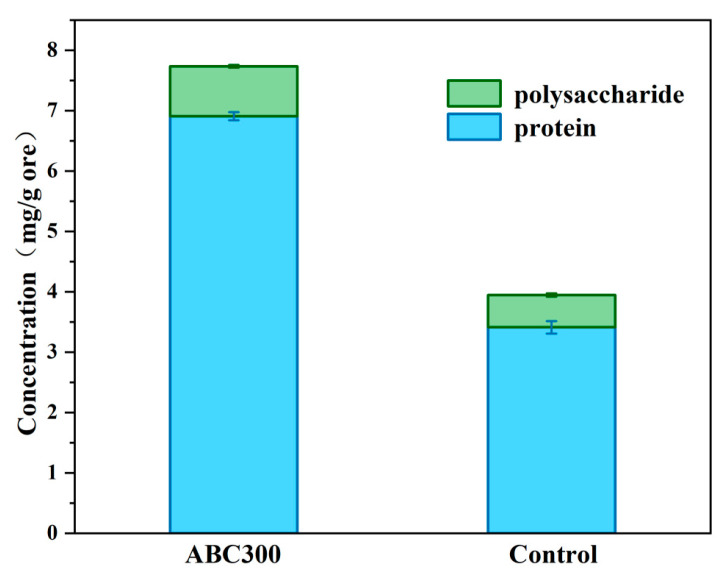
Protein and polysaccharide contents of EPS on the surface of the biopassivated ore samples.

As shown in Figure 11, the protein content and proportion within the EPS of the experimental group significantly increased, exceeding those of the control group. Data suggest that an elevated protein/polysaccharide ratio is beneficial for the attachment of EPS to the cell surface and the stability of biofilms [54]. Moreover, the polysaccharide content in the EPS of the experimental group was greater than that of the control group. The secretion of extracellular polysaccharides induced by biochar particles is regarded as a protective behavior of cells [55]. The increase in extracellular polysaccharides promotes cell aggregation and facilitates electron transfer within the EPS. It is evident that algal biochar promotes the synthesis of EPS and increases the protein-to-polysaccharide ratio in EPS, thereby increasing the synthesis and stability of the biofilm and electron transfer in the biofilm, which in turn, facilitates the formation of passivation layers.

### 3.7. Mechanisms by Which Algal Biochar Promotes Biopassivation

**Figure 12:**
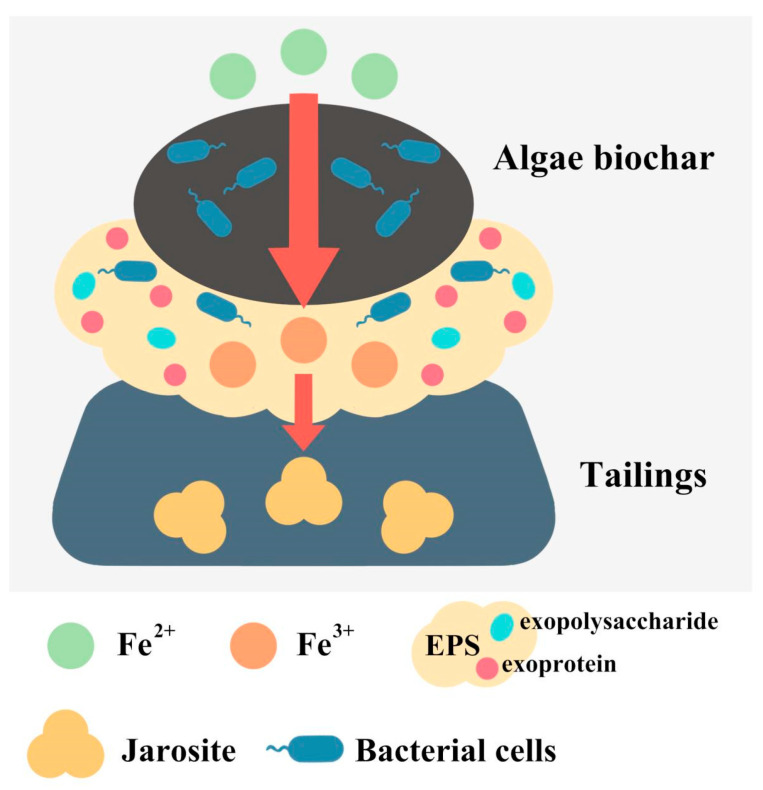
Mechanism of action of biochar for the biopassivation of tailings.

Based on the above results, the mechanism by which algal biochar prepared from *Limnospira maxima* enhances the biopassivation of copper sulfide tailings is summarized in Figure 12. The effects of algal biochar within the biopassivation system primarily include (1) adjusting the pH and ORP of the passivation system, (2) serving as a framework to adsorb free *A. ferrooxidans*, (3) adsorbing and enriching metal cations in the solution, (4) stimulating the production of EPS and the formation of biofilms, and (5) facilitating electron transfer during the passivation process. Specifically, its porous structure is particularly rich in oxygen-containing functional groups, such as -COOH and -OH, which establish a dual-functional interface. This interface immobilizes *A. ferrooxidans* through electrostatic interactions and sequesters metal cations via complexation/ion exchange mechanisms. This spatial co-localization fosters microreactors that enhance microbial-mineral interfacial reactions. Concurrently, the graphitic structure facilitates electron transfer between microbial cells and mineral surfaces, promoting Fe^2+^ oxidation kinetics and the subsequent precipitation of iron oxides. It also maintains system pH and ORP through proton buffering capacity. Furthermore, the adsorbed metal ions stimulate quorum-sensing-mediated EPS biosynthesis, with the carbon matrix serving as a scaffold for three-dimensional biofilm development. The resulting biofilm architecture consolidates the passivation layer. These effects further promote the formation and stability of the passivation layer, ultimately inhibiting mineral dissolution.

## 4. Conclusions

In this study, a novel method utilizing algal biochar to enhance the biopassivation of copper sulfide tailings was proposed. The effects of pyrolysis temperature and algal biochar concentration on the passivation efficacy and stability were examined. It was discovered that the introduction of algal biochar markedly facilitated the passivation of copper sulfide tailings by *A. ferrooxidans* and concurrently improved the stability of the passivation layer, effectively suppressing the acidification of copper sulfide tailings and preventing the generation of acid mine drainage. Algal biochar promoted the formation of a passivation layer and inhibited mineral dissolution by regulating the pH and ORP of the system, adsorbing to cells, enriching metal cations in the solution, increasing the production of extracellular polymeric substances, and enhancing electron transfer. The aforementioned findings have filled the gap in existing research on the promotion of biopassivation by algal biochar, while further exploring the stability issues within current biopassivation studies, making them more applicable to practical use. The experimental results indicated that a pyrolysis temperature of 300 °C and an algal biochar concentration of 6 g/L significantly promoted biopassivation and enhanced the stability of the passivation layer.

## Figures and Tables

**Table 1:** XRF analysis of major elements in the original ore sample.

Name	(%)
O	50.23
Si	26.01
Al	12.5
S	6.141
K	2.466
Fe	1.608
Cu	0.1697

**Table 2:** Significance analysis of pH and total copper concentration in groups with different biochar additions at the conclusion of the biopassivation experiment.

Biochar	pH	Total Copper Concentration (mg/L)
CK	1.403 ± 0.006 ^c^	2.352 ± 0.047 ^a^
ABC300	1.563 ± 0.025 ^a^	1.742 ± 0.052 ^d^
ABC400	1.497 ± 0.012 ^b^	2.077 ± 0.025 ^c^
ABC500	1.423 ± 0.021 ^c^	2.186 ± 0.012 ^b^

Means within the same row with different superscript letters differ significantly at *p* < 0.05 as determined by one-way ANOVA.

**Table 3:** Significance analysis of pH and total copper concentration in groups under different concentrations of biochar addition at the conclusion of the biopassivation experiment.

ABC300	pH	Total Copper Concentration (mg/L)
0 g/L	1.403 ± 0.006 ^d^	2.352 ± 0.047 ^a^
2 g/L	1.563 ± 0.025 ^c^	1.742 ± 0.052 ^b^
4 g/L	1.600 ± 0.010 ^b^	1.705 ± 0.042 ^b^
6 g/L	1.620 ± 0.017 ^ab^	1.552 ± 0.007 ^c^
8 g/L	1.633 ± 0.012 ^ab^	1.521 ± 0.001 ^c^
10 g/L	1.643 ± 0.006 ^a^	1.500 ± 0.010 ^c^

Means within the same row with different superscript letters differ significantly at *p* < 0.05 as determined by one-way ANOVA.

**Table 4:** Significance analysis of pH and total copper concentration in groups with different biochar additions at the conclusion of the simulated precipitation experiment.

Biochar	pH	Total Copper Concentration (mg/L)
CK	3.000 ± 0.026 ^d^	0.645 ± 0.014 ^a^
ABC300	3.437 ± 0.015 ^a^	0.337 ± 0.016 ^c^
ABC400	3.347 ± 0.015 ^b^	0.558 ± 0.007 ^b^
ABC500	3.147 ± 0.015 ^c^	0.582 ± 0.004 ^b^

Means within the same row with different superscript letters differ significantly at *p* < 0.05 as determined by one-way ANOVA.

**Table 5:** Significance analysis of pH and total copper concentration in groups under different concentrations of biochar addition at the conclusion of the simulated precipitation experiment.

ABC300	pH	Total Copper Concentration (mg/L)
0 g/L	3.000 ± 0.026 ^d^	0.645 ± 0.014 ^a^
2 g/L	3.437 ± 0.015 ^c^	0.337 ± 0.016 ^b^
4 g/L	3.553 ± 0.015 ^b^	0.256 ± 0.006 ^c^
6 g/L	3.677 ± 0.015 ^a^	0.063 ± 0.003 ^e^
8 g/L	3.537 ± 0.023 ^b^	0.074 ± 0.001 ^de^
10 g/L	3.453 ± 0.015 ^c^	0.089 ± 0.002 ^d^

Means within the same row with different superscript letters differ significantly at *p* < 0.05 as determined by one-way ANOVA.

**Table 6:** EDS analysis of the original ore sample.

Element	Wt%	Wt% Sigma	Atomic %
O	67.14	0.13	81.58
S	19.58	0.09	11.87
K	12.94	0.08	6.43
Fe	0.35	0.08	0.12
Cu	0	0.13	0

**Table 7:** EDS analysis of the biopassivated ore sample without ABC300 addition.

Element	Wt%	Wt% Sigma	Atomic %
O	77.40	0.22	91.17
S	4.42	0.09	2.60
K	0.70	0.06	0.34
Fe	17.49	0.21	5.90
Cu	0.00	0.27	0.00

**Table 8:** EDS analysis of the biopassivated ore sample with ABC300 addition.

Element	Wt%	Wt% Sigma	Atomic %
O	49.52	0.21	74.69
S	9.62	0.11	7.24
K	2.28	0.07	1.41
Fe	38.57	0.22	16.66
Cu	0.00	0.28	0.00

**Figure A1:**
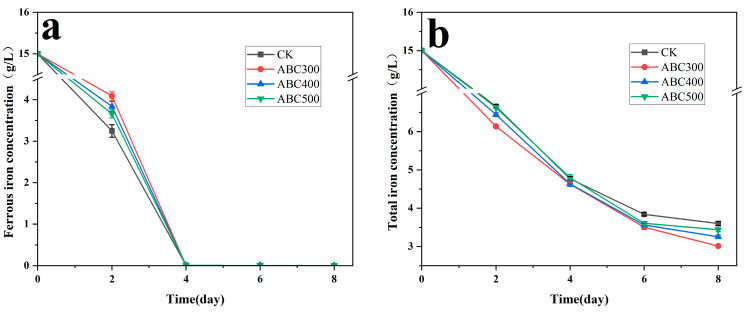
Changes in (**a**) ferrous iron concentration and (**b**) total iron concentration with different biochar addition amounts during the biopassivation experiments.

**Figure A2:**
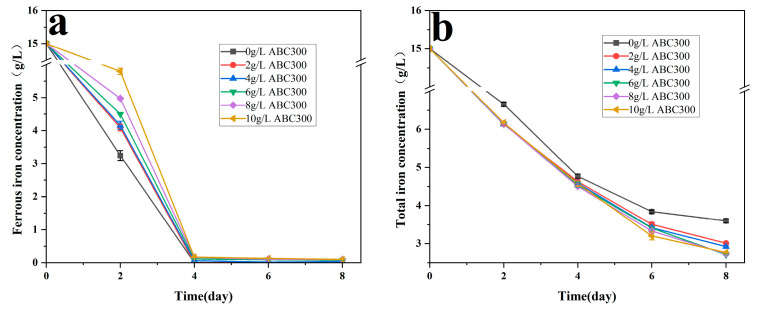
Changes in (**a**) ferrous iron concentration and (**b**) total iron concentration under different concentrations of biochar addition during the biopassivation experiments.

**Figure A3:**
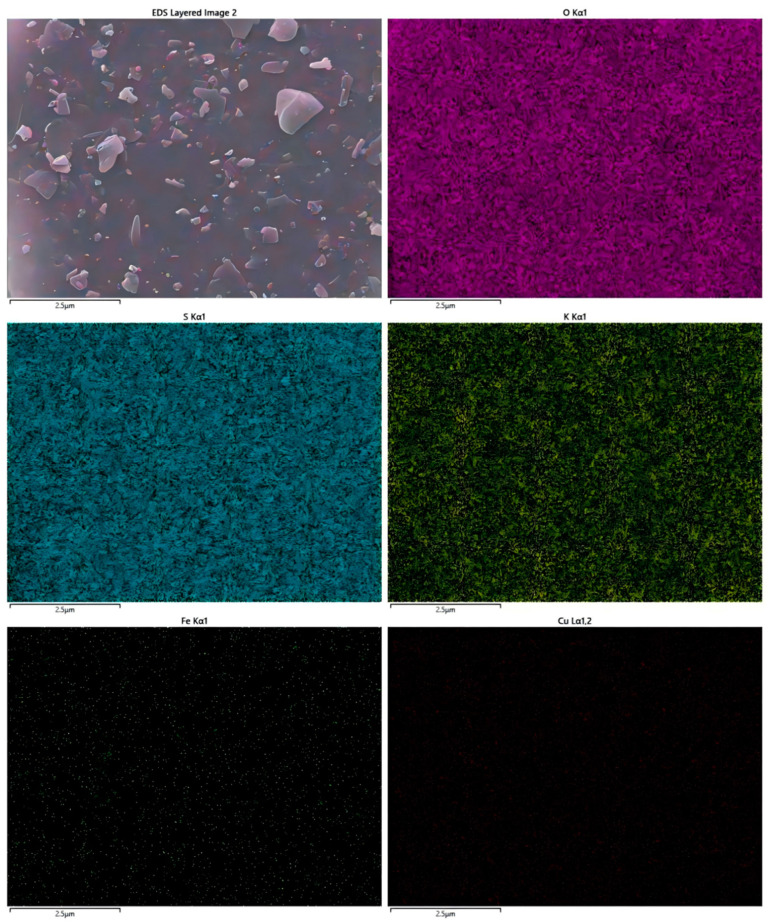
EDS results for the original ore sample.

**Figure A4:**
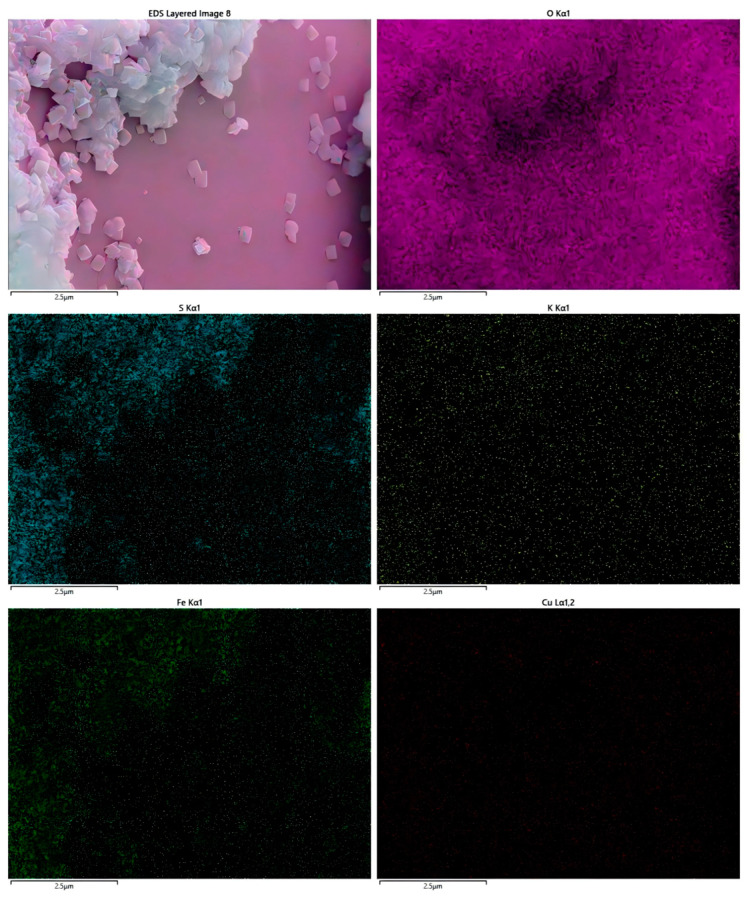
EDS results for the biopassivated ore sample without ABC300 addition.

**Figure A5:**
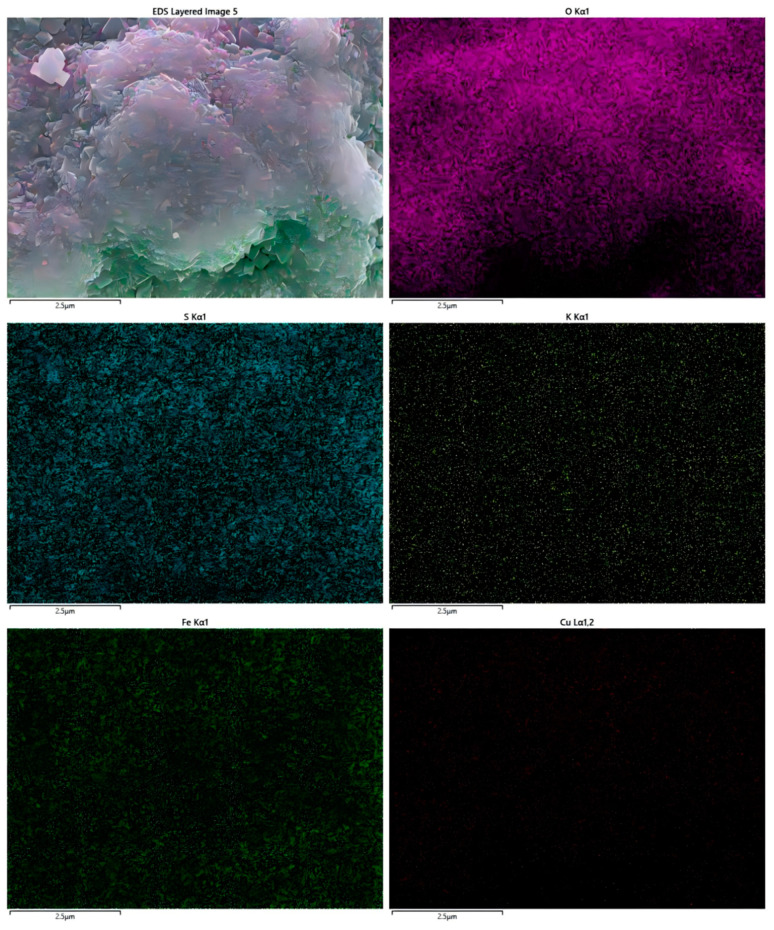
EDS results for the biopassivated ore sample with ABC300 addition.

## Data Availability

The original contributions presented in the study are included in the article material; further inquiries can be directed to the corresponding author.
